# Maternal cardiovascular health in early pregnancy and the risk of congenital heart defects in offspring

**DOI:** 10.1186/s12884-024-06529-5

**Published:** 2024-04-26

**Authors:** Dan-wei Zhang, Yi-bing Zhu, Si-jia Zhou, Xiu-hua Chen, Hai-bo Li, Wen-juan Liu, Zheng-qin Wu, Qiang Chen, Hua Cao

**Affiliations:** 1 Department of Cardiac Surgery, Fujian Children’s Hospital (Fujian Branch of Shanghai Children’s Medical Center), College of Clinical Medicine for Obstetrics & Gynecology and Pediatrics, Fujian Medical University, No.966 Hengyu Road, Jinan District, Fuzhou, 350014 People’s Republic of China; 2 Division of Birth Cohort Study, Fujian Maternity and Child Health Hospital, College of Clinical Medicine for Obstetrics & Gynecology and Pediatrics, Fujian Medical University, Fuzhou, People’s Republic of China; 3 Division of Birth Cohort Study, Fujian Children’s Hospital, Fuzhou, People’s Republic of China; 4 Division of Birth Cohort Study, Fujian Obstetrics and Gynecology Hospital, Fuzhou, People’s Republic of China

**Keywords:** Maternal cardiovascular health (CVH), Congenital heart disease (CHD), Maternal-fetal relations, Pregnancy, Heart disease risk factors, Birth cohort

## Abstract

**Background:**

Congenital heart disease (CHD) is the predominant birth defect. This study aimed to explore the association between maternal cardiovascular health (CVH) and the CHD risk in offspring.

**Methods:**

We used the prospective data from the Fujian Birth Cohort Study, collected from March 2019 to December 2022 on pregnant women within 14 weeks of gestation. Overall maternal CVH was assessed by seven CVH metrics (including physical activity, smoking, sleep duration, body mass index, blood pressure, total cholesterol, and fasting plasma glucose), with each metric classified as ideal, intermediate or poor with specific points. Participants were further allocated into high, moderate and low CVH categories based on the cumulative CVH score. The association with offspring CHD was determined with log-binominal regression models.

**Results:**

A total of 19810 participants aged 29.7 (SD: 3.9) years were included, with 7846 (39.6%) classified as having high CVH, 10949 (55.3%) as having moderate CVH, and 1015 (5.1%) as having low CVH. The average offspring CHD rate was 2.52%, with rates of 2.35%, 2.52% and 3.84% across the high, moderate and low CVH categories, respectively (*P* = 0.02). Adjusted relative risks (RRs) of having offspring CHD were 0.64 (95% CI: 0.45-0.90, *P* = 0.001) for high CVH and 0.67 (95% CI: 0.48-0.93, *P* = 0.02) for moderate CVH compared to low CVH. For individual metrics, only ideal total cholesterol was significantly associated with lower offspring CHD (RR: 0.73, 95% CI: 0.59-0.83, *P* = 0.002).

**Conclusions:**

Pregnant women of high or moderate CVH categories in early pregnancy had reduced risks of CHD in offspring, compared to those of low CVH. It is important to monitor and improve CVH during pre-pregnancy counseling and early prenatal care.

**Supplementary Information:**

The online version contains supplementary material available at 10.1186/s12884-024-06529-5.

## Brief synopsis

Mothers of better cardiovascular health (CVH) categories in early pregnancy showed reduced risks of having children with congenital heart disease (CHD), compared to those of low CVH. Integrating maternal CVH monitoring and risk management into prenatal care is essential for improving pregnancy outcomes.

## Background

Congenital heart defect (CHD) is the most common congenital anomaly in the world [1]. In China, the prevalence of CHD in 2020 was 17.32 cases per 1000 perinatal births [2]. The lack of understanding of the modifiable risk factors of CHD has hindered its prevention [3]. Cardiovascular disease complicates nearly 1–4% of pregnancies. Moreover, recent studies indicate that maternal cardiovascular health (CVH) may impact offspring health, and acknowledges the cruciality of the window of gestation and surrounding periods [4, 5].

The American Heart Association (AHA) has proposed eight factors to measure CVH, which include diet, physical activity (PA), nicotine exposure, sleep health, body mass index (BMI), blood lipids, blood glucose, and blood pressure (BP) [6]. Certain factors, such as overweight or obesity [7, 8], and diabetes [9, 10], are known to increase the risk of CHD or other congenital anomalies in offspring. However, the effects of other factors, such as exercise during pregnancy, remained controversial [11, 12]. Increasing studies have begun to investigated the effect of combined metrics on pregnancy outcomes beyond birth defects. For example, some suggest that adherence to more favorable CVH is associated with lower risks of gestational diabetes and preeclampsia [13, 14], and is also beneficial to offspring health, including newborn birthweight and adolescent CVH [5, 15]. Nevertheless, the joint effect of maternal overall CVH on CHD development in offspring remains unclear.

Accordingly, this study aimed to examine the association between early pregnancy CVH status of pregnant women and the incidence of CHD in their offspring, using data from the Fujian Birth Cohort Study in southeast China. We first identified seven metrics to indicate the overall CVH, and the participants were classified as having high, moderate or low CVH. We then assessed the relative risk (RR) of offspring CHD across different CVH levels. The influence of individual CVH metrics on offspring CHD were also examined, and multiple sensitivity analyses were conducted to validate our findings.

## Methods

### Study design and population

The Fujian Birth Cohort Study is a large, ongoing, prospective cohort study designed to investigate potential risk factors for birth defects during pregnancy [16]. Pregnant women who are within 14 weeks of gestation, plan to attend routine prenatal examinations and give birth at the study site, are eligible. All participants give their informed written consent and have an in-person baseline interview when enrolled. Then, they are followed up at 22–26 weeks, 32-36 weeks of gestation, at delivery and 42 days after delivery.

**Fig. 1:**
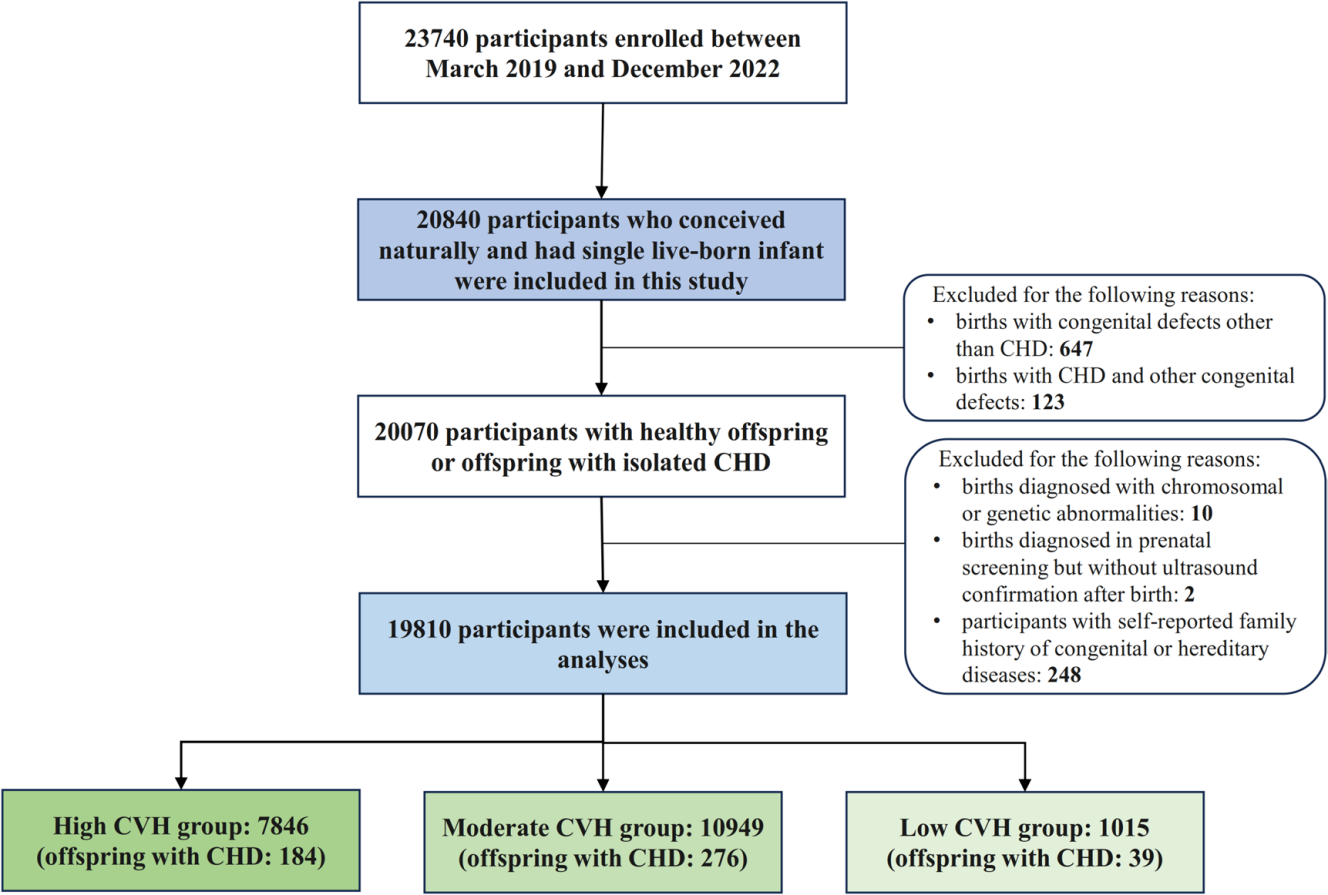
Study flow chart. The flow chart shows detailed inclusion and exclusion criteria for this study as well as the number of participants. CHD: congenital heart disease; CVH: cardiovascular health

A total of 23740 participants were enrolled, interviewed and had their pregnancy outcomes documented from March 2019 to December 2022. In this study, we included 20840 participants who conceived naturally and had a live-born singleton, since multiple pregnancies or pregnancies conceived via assisted reproductive technology may increase the risk of offspring CHD. We first excluded births presenting with other congenital anomalies (*n* = 647), or a combination of CHD and other congenital anomalies (*n* = 123). Further exclusions were made for births diagnosed with chromosomal or genetic abnormalities (*n* = 10), those without ultrasound confirmation after birth (*n* = 2), and participants reporting a family history (within three generations) of congenital or hereditary diseases (*n* = 248). Finally, 19810 participants were included (flowchart in Fig. 1).

### Maternal CVH

Maternal CVH was characterized using the data in baseline interview. It was a combination of three health behaviors (PA, smoking, and sleep health) and four clinical indicators (BMI, BP, blood lipid, and blood glucose). Health-related information was prospectively collected by questionnaires, and specific biological samples were collected. Height, weight, and BP were measured by trained personnel, and BMI was calculated as weight (kg)/height^2^ (m^2^). The method for processing and preserving biological samples have been described previously [16].

Each CVH metric was classified as ideal, intermediate, or poor according to available guidelines or prior studies, [17–19] and was assigned 2 points for ideal, 1 point for intermediate, and 0 points for poor or nonideal. The detailed classification of CVH metrics was listed in Table 1. The overall CVH score was calculated by summing seven metrics, and was categorized as high [12-14 points], moderate [9-11 points] or low CVH [0-8 points].

**Table 1:** Definitions of individual maternal cardiovascular health (CVH) metrics

**CVH metric**	**Ideal**	**Intermediate**	**Poor**
**Physical activity (PA)**	Moderate or vigorous PA per week	Low-intensity PA per week	Without any PA
**Smoke**	Never or quit > 12 months	Quit ≤ 12 months	Current smoker
**Sleep health**	Sleep duration ≥ 7 h and < 9 h	Sleep duration ≥ 5 h and < 7 h, or ≥ 9 h	Sleep duration < 5 h
**Body mass index (BMI)**^a^	BMI ≥ 18.5 and < 25 before pregnancy, and a maximal gestational weight gain of 2 kg in early pregnancy	All others “nonideal”	
**Blood pressure (BP)**^b^	No history of hypertension, and the systolic blood pressure (SBP) < 120 mmHg and the diastolic blood pressure (DBP) < 80 mmHg	SBP ≥ 120 and < 140 mmHg, or DBP ≥ 80 and < 90 mmHg	A history of hypertension, or SBP ≥ 140 mmHg or DBP ≥ 90 mmHg
**Blood lipid**	Total cholesterol of (TC) < 200 mg/dL	TC ≥ 200 and < 240 mg/dL	TC ≥ 240 mg/dL
**Blood glucose**	No history of diabetes, and the fasting plasma glucose (FPG) < 100 mg/dL	FPG ≥ 100 and < 126 mg/dL	A history of diabetes, or FPG ≥ 126 mg/d

*BMI* body mass index, *BP* blood pressure, *CHD* congenital heart disease, *CVH* cardiovascular health, *DBP* diastolic blood pressure, *FPG* fasting plasma glucose, *PA* physical activity, *SBP* systolic blood pressure, *TC* total cholesterol

^a^BMI = weight (kg)/(height [m])^2^

^b^We defined the BP metrics according to Chinese guideline

### Offspring CHD

The outcome was CHD in live-born singletons. The CHD cases were diagnosed using the International Classification of Diseases version 10 (ICD-10) ‘Q20-Q26’ and reported to the National Maternal and Child Health Monitoring System, following the guidelines outlined in operation manual of the National Maternal and Child Health Surveillance [20]. Specifically, the CHD was initially identified by ultrasound scans during a designated postnatal monitoring period, and confirmed by a team of specialized pediatric cardiologists, ultrasound technicians, and obstetricians. According to the reporting criteria [20, 21], the following minor abnormalities were not identified as CHD: (1) isolated patent ductus arteriosus or patent foramen ovale in preterm infants; (2) isolated diameters of pulmonary artery end or patent foramen ovale less than 3 mm in full-term infants 24 h after birth; and (3) isolated mild tricuspid valve regurgitation.

### Confounding variables

Confounding variables were obtained via self-report questionnaires, including maternal demographics (i.e., education [below college, college or above], marriage [married, single or divorced], monthly income [≤ 1500, 1500-9000, ≥ 9000 yuan], occupation [employed, self-employed, unemployed]), health information (i.e., drinking, age of menarche, medication use around this pregnancy, history of chronic disease such as hypertension, diabetes, thyroid diseases, and breast or gynecological diseases), and pregnancy history (i.e., previous pregnancies, pregnancy comorbidities or complications, abnormal pregnancy outcomes).

### Statistical analysis

We compared the characteristics among different maternal CVH categories, with the χ2 test for categorical variables and the Kruskal-Wallis test for continuous variables.

In the primary analysis, we investigated the association between maternal CVH categories and the risk of CHD in offspring by building a series of log-binomial regression models. The relative risk (RR) was computed as a measure of risk, adjusting for maternal demographics and several key variables (drinking, medication use, previous pregnancies, and chronic diseases). We also used the inverse probability of treatment weighting (IPTW) approach to address all the confounding variables mentioned above. Moreover, the population attributable fraction of CVH status was calculated [22, 23]. In the secondary analyses, we further evaluated the association of individual CVH metrics with CHD in offspring. When performing the analyses on individual CVH metrics, we combined the intermediate or poor levels as a single nonideal category for each metric, and the other metrics were also adjusted.

Multiple sensitivity analyses were conducted to validate primary findings. Firstly, we examined the association of overall CVH score and CHD in offspring, restricted cubic spline analysis was also used. Secondly, since the CHD rate was low, the logistic regression approach was conducted to repeat the primary analyses, and odds ratios (ORs) were calculated. Thirdly, we used non-high-density lipoprotein cholesterol (non-HDL-C, ideal: < 130 mg/dL, intermediate: 130-160 mg/dL, poor: > 160 mg/dL) as the indicator of blood lipids instead of TC. Lastly, given that there were controversies regarding PA recommendations in early pregnancy, we excluded PA and reclassified the CVH categories (high [11-12 points], moderate [8-10 points] and low CVH [0-7 points]).

All statistical tests were two sided, with a significance level of 0.05. We performed all the analyses using SAS 9.4 (SAS Institute Inc., Cary, NC).

## Results

### Population characteristics

There were 19810 participants included, with a mean age of 29.7 (standard deviation [SD]: 3.9) years. A total of 7846 (39.6%) participants were classified as having high CVH (mean CVH score: 12.4 [SD: 0.55]), 10949 (55.3%) as having moderate CVH (10.2 [SD: 0.77]), and 1015 (5.1%) as having low CVH (7.76 [SD: 0.52]). Most characteristics were different among the three CVH categories (Table 2). Participants with higher CVH had greater proportions of having better education (High: 84.0%; Moderate: 78.1%; Low: 69.9%; *P* < 0.001) and higher income (High: 18.8%; Moderate: 16.4%; Low: 15.4%; *P* < 0.001). They also showed lower proportions of having previous history of adverse pregnancy outcomes (High: 28.4%; Moderate: 29.8%; Low: 31.3%; *P* = 0.04) and less medication use around pregnancy (High: 45.0%; Moderate: 47.1%; Low: 51.1%; *P* < 0.001).

**Table 2:** Population characteristics among pregnant women with different cardiovascular health (CVH) statuses

	**Total (*n* = 19,810)**	**High CVH (*n* = 7846)**	**Moderate CVH (*n* = 10,949)**	**Low CVH (*n* = 1015)**	***P* value**
Age, years, mean (SD)	29.7 (3.9)	29.8 (3.7)	29.6 (4)	29.8 (4.4)	< 0.0001
College or above, n (%)	15850 (80.0)	6587 (84.0)	8554 (78.1)	709 (69.9)	< 0.0001
Married, n (%)	18723 (94.5)	7518 (95.8)	10263 (93.7)	942 (92.8)	< 0.0001
Employed, n (%)	10011 (50.5)	3956 (50.4)	5562 (50.8)	493 (48.6)	0.38
Income, n (%)					
low	3792 (19.1)	1310 (16.7)	2222 (20.3)	260 (25.6)	< 0.0001
moderate	12585 (63.5)	5059 (64.5)	6927 (63.3)	599 (59.0)	0.002
high	3433 (17.3)	1477 (18.8)	1800 (16.4)	156 (15.4)	< 0.0001
Never drink, n (%)	17120 (86.4)	6724 (85.7)	9502 (86.8)	894 (88.1)	0.029
Never smoke, n (%)	19403 (97.9)	7783 (99.2)	10681 (97.6)	939 (92.5)	< 0.0001
**Clinical health indicator, mean (SD)**					
BMI before pregnancy, kg/m^2^*	21.1 (2.8)	21 (1.7)	21 (3.2)	22.2 (4.3)	< 0.0001
BMI in the early pregnancy, kg/m^2^*	21.4 (2.9)	21.1 (1.8)	21.4 (3.3)	22.9 (4.1)	< 0.0001
SBP, mmHg	114 (10.6)	111.0 (9.5)	115.6 (10.7)	123.1 (9.8)	< 0.0001
DBP, mmHg	68.8 (8.4)	66.9 (7.6)	69.8 (8.5)	73.9 (9.0)	< 0.0001
FPG, mg/dL	85.4 (6.9)	84.9 (5.6)	85.5 (6.9)	89.0 (12.8)	< 0.0001
TC, mg/dL	179.2 (27.2)	171.9 (21.8)	181.8 (27.7)	206.8 (35.1)	< 0.0001
Non-HDL-C, mg/dL	114 (23.7)	108.0 (18.9)	116.2 (24.3)	138.0 (30.5)	< 0.0001
**Disease history, n (%)**					
hypertension	15 (0.1)	0 (0.0)	9 (0.1)	6 (0.6)	< 0.0001
diabetes	23 (0.1)	1 (0.0)	13 (0.1)	9 (0.9)	< 0.0001
hyperthyroidism	128 (0.6)	51 (0.7)	72 (0.7)	5 (0.5)	0.82
other thyroid disease	48 (0.2)	27 (0.3)	20 (0.2)	1 (0.1)	0.054
breast and gynecological diseases	726 (3.7)	329 (4.2)	368 (3.4)	29 (2.9)	0.004
**Pregnancy history, n (%)**					
previous pregnancies	11246 (56.8)	4553 (58.0)	6112 (55.8)	581 (57.2)	0.010
previous births	8151 (41.1)	3362 (42.8)	4379 (40.0)	410 (40.4)	< 0.001
abnormal pregnancy outcomes	5811 (29.3)	2230 (28.4)	3263 (29.8)	318 (31.3)	0.044
pregnancy complications	1618 (8.2)	643 (8.2)	888 (8.1)	87 (8.6)	0.87
Periconceptional medical use, n (%)	9209 (46.5)	3529 (45.0)	5161 (47.1)	519 (51.1)	< 0.001
**Pregnancy outcomes**					
gestational weeks, week, mean (SD)	39.2 (1.4)	39.3 (1.4)	39.2 (1.4)	39.0 (1.6)	< 0.0001
cesarean section, n (%)	6674 (33.7)	2526 (32.2)	3728 (34.0)	420 (41.4)	< 0.0001
offspring gender, boy, n (%)	10290 (51.9)	4000 (51.0)	5765 (52.7)	525 (51.7)	0.13
offspring weight, g, mean (SD)	3246.4 (431.4)	3254.9 (420.6)	3239.1 (432.4)	3259.9 (494.4)	0.008

*BMI* body mass index, *CHD* congenital heart disease, *CVH* cardiovascular health, *DBP* diastolic blood pressure, *FPG* fasting plasma glucose, *Non-HDL-C* non-high density lipoprotein cholesterol, *PA* physical activity, *SBP* systolic blood pressure, *SD* standard deviation, *TC* total cholesterol

^*^BMI = weight (kg)/(height [m])^2^. The weight and height before pregnancy were collected by questionnaire, and weight in early pregnancy was measured during the first interview in early pregnancy

### Maternal CVH and CHD in offspring

The median maternal overall CVH score was 11 (inter-quartile range [IQR]: 10-12). The distribution of overall CVH score is shown in Supplemental Figure S1. For each CVH metric, the proportions of participants at ideal, intermediate, and poor levels are shown in Supplemental Figure S2. A total of 499 (2.52%) participants had offspring diagnosed with CHD; the prevalence of offspring CHD was 2.35% in the high CVH category, slightly higher in the moderate CVH category at 2.52%, and highest in the low CVH category at 3.84% (*P* = 0.02). For each level of individual CVH metrics, the rate of CHD in offspring is listed in Supplemental Table S1.

### Primary analysis

The relationship between maternal CVH category in early pregnancy and CHD in offspring is shown in Table 3 and Supplemental Table S2. The unadjusted RR was 0.61 (95% confidence interval [CI]: 0.43-0.86, *P* = 0.004) for pregnant women in high CVH category and 0.66 (95% CI: 0.47-0.91, *P* = 0.012) for moderate CVH category, compared to pregnant women in low CVH category (Supplemental Table S2). After adjustment, the RRs slightly increased to 0.64 (95% CI: 0.45-0.90, *P* = 0.01) and 0.67 (95% CI: 0.48-0.93, *P* = 0.02) for the high and moderate CVH categories, respectively, compared to low CVH (Table 3). The results remained consistent in the IPTW analysis, as the RR was 0.66 (95% CI: 0.47-0.93, *P* = 0.02) for high CVH and 0.68 (95% CI: 0.49-0.95, *P* = 0.03) for moderate CVH (Supplemental Table S2). The population attributable fraction for the moderate and poor CVH on offspring CHD in relation to high CVH was estimated as 4.5% (Supplemental Table S3).

**Table 3:**
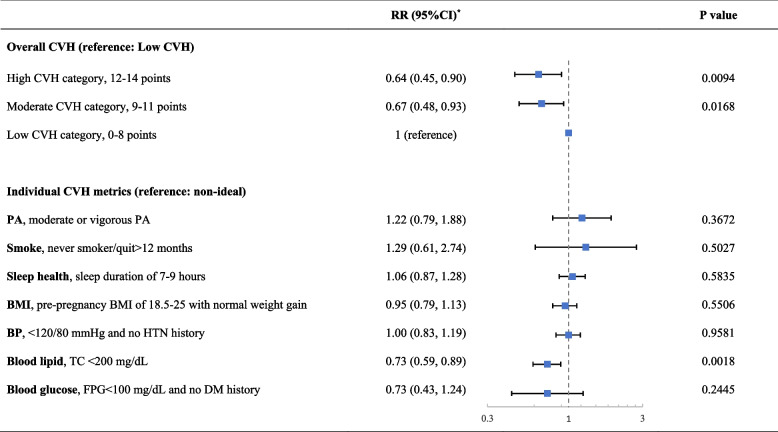
The association of maternal cardiovascular health (CVH) with congenital heart disease (CHD) in offspring

*BMI* body mass index, *CHD* congenital heart disease, *CI* confidence interval, *CVH* cardiovascular health, *FPG* fasting plasma glucose, *PA* physical activity, *RR* relative risk, *TC* total cholesterol

^*^The relative risk is computed from the log-binominal regression model, with the adjustment of key confounding variables including maternal demographics, drinking, medication use, previous pregnancies, and chronic diseases; for each individual CVH metrics, other metrics are further adjusted

### Secondary analyses

The Table 3 and Supplemental Table S4 show the association between individual CVH metrics (defined as ideal vs nonideal) and offspring CHD. Among these metrics, only an ideal TC was significantly associated with lower CHD in offspring, and the RR was 0.73 (95% CI: 0.59-0.83, *P* = 0.002), adjusting for other metrics and confounding factors. Specifically, when examined for exact values (Supplemental Table S4), higher BMI (RR: 1.04, 95% CI: 1.01-1.07, *P* = 0.02), TC (RR: 1.01, 95% CI: 1.00–1.01, *P* = 0.001) and FPG (RR: 1.01, 95% CI: 1.00–1.02, *P* = 0.012) were independently associated with higher risks of CHD in offspring, while longer sleep duration was associated with lower CHD (RR: 0.91, 95% CI: 0.83-0.99, *P* = 0.024).

### Sensitivity analyses

For the continuous CVH score, the RR of CHD in offspring was 0.95 (95% CI: 0.90-1.01, *P* = 0.10) for every one-point increase (Supplemental Table S2); the restricted cubic spline also illustrated a trend of lower risk of offspring CHD for higher CVH score, though it did not achieve a statistical significance (Supplemental Figure S3). In multivariable logistic regression analyses, the ORs of high and moderate CVH were 0.63 (95% CI: 0.44-0.89) and 0.66 (95% CI: 0.47-0.93) compared to low CVH, respectively (Supplemental Table S5). After replacing TC with non-HDL-C (Supplemental Table S6), the number of participants in each CVH category was 7737 (High, CHD: 2.4%), 10940 (Moderate, CHD: 2.5%) and 1133 (Low, 3.6%), with the RRs being 0.68 (95% CI: 0.49-0.96, High vs Low) and 0.70 (95% CI: 0.51-0.97, Moderate vs Low), respectively. After excluding PA from the overall CVH (Supplemental Table S7), the number of participants in each CVH category was 9526 (High, CHD: 2.3%), 9837 (Moderate, CHD: 2.6%) and 447 (Low, 4.9%), and the RRs were 0.48 (95% CI: 0.31-0.74, High vs Low) and 0.54 (95% CI: 0.35-0.83, Moderate vs Low), respectively.

## Discussion

Our study revealed that pregnant women in high or moderate CVH categories had lower risks of CHD in their offspring than those in low CVH category. The cumulative effect of overall CVH outweighed the impact of single CVH metrics, indicating the importance of a comprehensive management of overall CVH. The findings provide instructive measures for risk communication and early prevention of offspring CHD around pregnancy.

Emerging evidence has emphasized the importance of lifetime CVH, yet studies about gestational CVH are limited, with interested pregnancy outcomes varying across studies [5, 14, 24]. To our knowledge, the impact of maternal CVH on CHD in offspring remains unclear, and our study contributes to this area by thoroughly investigating the association. We measured the maternal CVH according to criteria from previous high-quality literature and computing the RR with sufficient adjustment [17, 18]. Seven critical health behaviors and physiological indicators in early pregnancy were incorporated in the CVH score, because the first trimester is the key stage for fetal heart development and maternal CVH status during this period is close to that before pregnancy. Some previous studies shared several factors with our study when defining CVH, and they demonstrated that better maternal CVH reduced multiple adverse outcomes in offspring, such as low birthweight and small-for-gestational-age [25], or later cardiovascular and metabolism problems [5]. We observed a lower risk of CHD in offspring in mothers with better CVH compared to those with extremely low CVH status; while in relation to the high CVH category, the adjusted population attributable fraction on offspring CHD related to other lower maternal CVH categories was 4.5%. The CVH status consisted of modifiable risk factors, and if all the pregnant women achieved high CVH status, then approximately nearly 23 cases could be prevented in this cohort. Furthermore, our findings also suggested that there were other factors that may lead to CHD in offspring. The etiology of CHD is multi-factorial as both genetic and environmental exposures contributing to its development [26]. Although we excluded offspring with possible or determined genetic causes in order to study the independent effect of maternal CVH, we could not obtain the genetic information of every participant and their offspring. There are complex functional interactions between genomics variation and environmental exposures that modulate the critical biological system of heart development [27]. The role of epigenetics in CHD development has also become the focus of extensive research recently [28]. However, as the cause and mechanism for most CHD remain unknown, our research offers a practical approach towards preventing CHD at this stage.

Although limited studies have explored the association between the maternal overall CVH and CHD in offspring, our findings are aligned with what we expected given that several individual CVH metrics were previously implicated as risk factors for offspring CHD. The novel aspect of our research is that a combination of these metrics may provide more than additive effects. Our primary result indicated that mothers of high CVH category could eliminate nearly one-third of the offspring CHD than those of low CVH category. Our secondary analyses were in line with previous studies, indicating that the risk of offspring CHD increased with higher maternal BMI [7, 29] blood glucose [9, 10], and blood lipids [30, 31], while decreased with longer sleep duration. When the comparison was made between the ideal and nonideal groups instead of continuous values, there was probably a lack of sufficient power to uncover a significant effect since most of the participants fell within the ideal range. PA during pregnancy is widely debated; traditional advice often suggest reducing levels of PA, but new investigations advocate for the benefits of maintaining regular PA. Our study observed that only a small proportion of the pregnant women had achieved moderate or vigorous PA, with no significant association of offspring CHD identified. In summary, we demonstrated that the overall CVH of pregnant women could exert a comprehensive and critical impact on offspring CHD, and we also suggested a holistic approach to overall maternal CVH monitoring over focusing on single metrics solely. This perspective could offer a more effective strategy for early risk management during pregnancy.

We found that pregnant women with low CVH were at the highest risk of having offspring with CHD, however, our results did not show marked differences between the moderate and high CVH categories, and the analysis based on continuous CVH score did not reach statistical significance. This outcome might be attributed to several factors. One could be that current used definition of CVH, which is widely accepted, might not be perfectly suitable for classifying pregnant women, particularly that our study population were of relatively young (mean age: 30 years). Besides, the interested outcome—CHD, is of low incidence, combined with the fact that most CHD cases occur in low risk pregnancies [32], which might further explain the lack of significance. Besides, a series of sensitivity analyses supported our primary findings. When we adjusted our modelling strategy to logistic regression model, or modified the definition of specific individual CVH, the low CVH category still showed the highest risk of CHD in the offspring, though effect size varied slightly. The consistency across various analyses reinforced the robustness of our primary findings, emphasizing the significance of overall CVH in the context of early pregnancy.

Our study has several clinical implications. Firstly, We strongly implied the necessity to conduct early screening, monitoring and intervention on CVH for pregnant women, even women of reproductive age, and we have provided an easy construct for CVH characterization. For low risk pregnancies [33], the assessment of overall CVH could be taken into account to provide a more comprehensive risk evaluation for CHD in offspring. Furthermore, for pregnant women identified with extremely poor CVH, a thorough fetal cardiac scan becomes necessary and critical [32, 34]. Additionally, a standardized and exacting standard for measuring CVH in pregnant women is urgently needed, along with methods for implementing CVH monitoring and risk modification.

There were some limitations in this study. First, if the CHD was diagnosed later than the follow-up period (typically within 30-42 days postpartum), we may miss the outcome. Second, limited guidelines existed in defining ideal CVH metrics during pregnancy, thus, we mainly referred to established high-quality publications; however, further studies concerning the CVH measurement and risk stratification specifically for pregnant women are required. Third, our study was conducted during the COVID-19 pandemic, but we could not obtain the infectious status of participants. It is noteworthy that there was no outbreak in Fujian province during that study period, moreover, previous studies have suggested that SARS-CoV-2 infection is unlikely to cause congenital anomalies [35].

## Conclusions

This study finds that pregnant women with high or moderate CVH categories have significantly lower risks of bearing offspring with CHD, compared to those with low CVH categories. It highlighted the necessity of screening, monitoring and adjusting overall CVH status before and during the early stage of pregnancy.

### Supplementary Information

**Supplementary Material 1.**

## Data Availability

The datasets analysed during the current study are not publicly available currently but are available from the corresponding author on reasonable request.

## Abbreviations

AHA: American Heart Association
BMI: Body mass index
BP: Blood pressure
CHD: Congenital heart disease
CI: Confidence interval
CVH: Cardiovascular health
DBP: Diastolic blood pressure
FPG: Fasting plasma glucose
HDL-C: High-density lipoprotein cholesterol
ICD: International Classification of Diseases
IPTW: Inverse probability treatment weighting
IQR: Interquartile range
PA: Physical activity
SBP: Systolic blood pressure
SD: Standard deviation
