# Supplementary material for: Maternal cardiovascular health in early pregnancy and the risk of congenital heart defects in offspring
Source: BMC Pregnancy Childbirth. 2024 Apr 26;24:325. doi: 10.1186/s12884-024-06529-5 (PMC11047036; doi:10.1186/s12884-024-06529-5)
Supplement: Supplementary file 1 — Supplementary Material 1. [file 12884_2024_6529_MOESM1_ESM.docx]

**SUPPLEMENTAL MATERIALS**

**Supplemental Figure S1.** Distribution of maternal overall CVH score

**Supplemental Figure S2.** The distribution of individual maternal CVH metrics.

**Supplemental Figure S3.** Restricted cubic spline for overall CVH score and odds of having offspring with CHD

**Supplemental Table S1.** The prevalence of CHD in offspring for pregnant women at different levels of individual CVH metrics

**Supplemental Table S2.** The association of maternal overall cardiovascular health (CVH) with congenital heart disease (CHD) in offspring

**Supplemental Table S3.** The population attributable fraction of maternal cardiovascular health (CVH) status on congenital heart disease (CHD) in offspring

**Supplemental Table S4.** The association of individual maternal cardiovascular health (CVH) metrics with congenital heart disease (CHD) in offspring

**Supplemental Table S5.** The association of maternal cardiovascular health (CVH) with congenital heart disease (CHD) in offspring (measured by logistic models)

**Supplemental Table S6.** The association of maternal cardiovascular health (CVH) with congenital heart disease (CHD) in offspring (non-high density lipoprotein cholesterol as lipid metric)

**Supplemental Table S7.** The association of maternal cardiovascular health (CVH) with congenital heart disease (CHD) in offspring (excluding the PA metric)

**Supplemental Figure S1. Distribution of maternal overall CVH score**

CVH, cardiovascular health


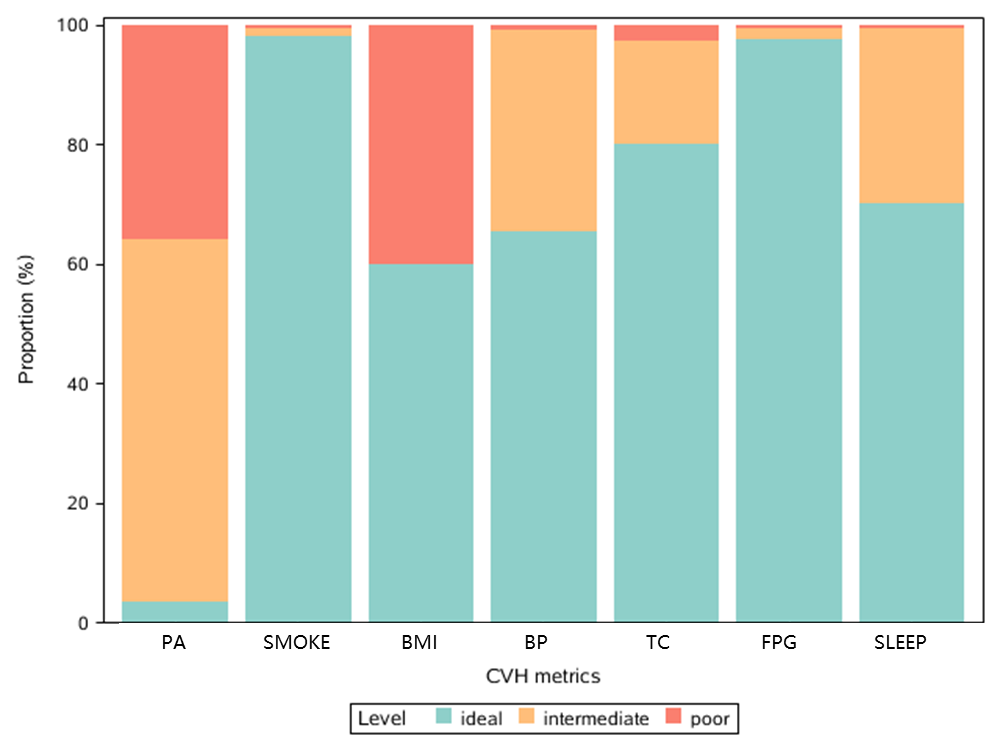


**Supplemental Figure S2. The distribution of individual maternal CVH metrics.**

BMI, body mass index; BP, blood pressure; CVH, cardiovascular health; FPG, fasting plasma glucose; PA, physical activity; TC, total cholesterol.


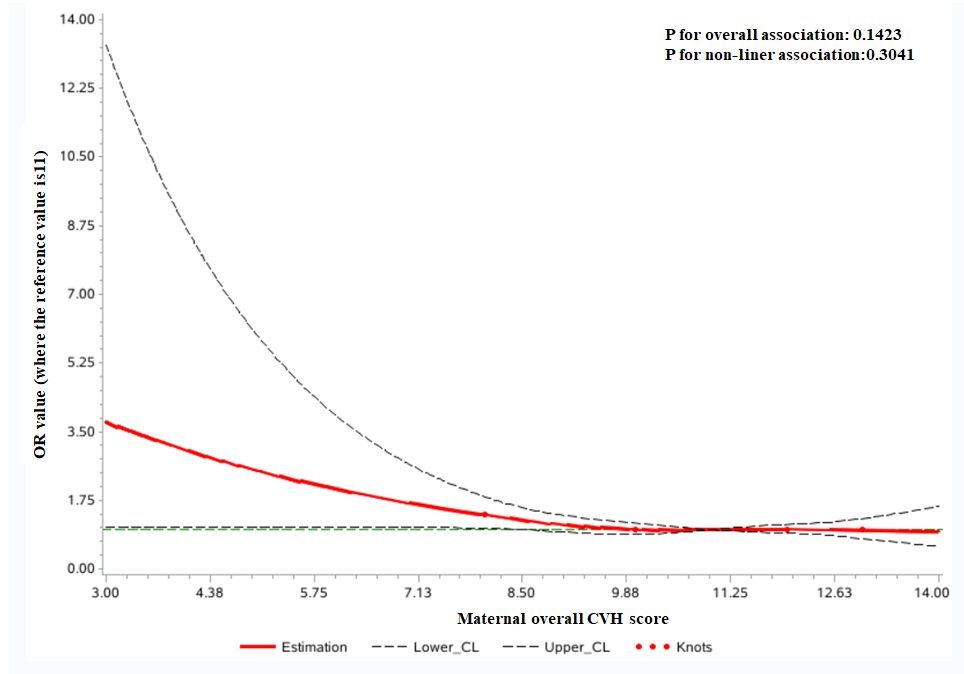


**Supplemental Figure S3. Restricted cubic spline for overall CVH score and odds of having offspring with CHD (reference: CVH score=11)**

CHD, congenital heart disease; CVH, cardiovascular health

**Supplemental Table S1.** **The prevalence of CHD in offspring for pregnant women at different levels of individual CVH metrics**

| **Individual CVH metrics** | **Ideal** | **Non-ideal** | |
| --- | --- | --- | --- |
|  |  | **Intermediate** | **Poor** |
| **PA** |  |  |  |
| number of participants, n | 714 | 12038 | 7058 |
| prevalence of offspring CHD, n (%) | 21 (2.9) | 300 (2.5) | 178 (2.5) |
| **Smoke** |  |  |  |
| number of participants, n | 19479 | 302 | 29 |
| prevalence of offspring CHD, n (%) | 492 (2.5) | 7 (2.3) | 0 (0.0) |
| **Sleep health, sleep duration** |  |  |  |
| number of participants, n | 13975 | 5787 | 48 |
| prevalence of offspring CHD, n (%) | 355 (2.5) | 143 (2.5) | 1 (2.1) |
| **BMI** |  |  |  |
| number of participants, n | 11956 | 7854 | |
| prevalence of offspring CHD, n (%) | 291 (2.4) | 208 (2.7) | |
| **BP** |  |  |  |
| number of participants, n | 13007 | 6691 | 112 |
| prevalence of offspring CHD, n (%) | 322 (2.5) | 174 (2.6) | 3 (2.7) |
| **Blood lipid, TC** |  |  |  |
| number of participants, n | 15946 | 3414 | 450 |
| prevalence of offspring CHD, n (%) | 374 (2.4) | 105 (3.1) | 20 (4.4) |
| **Blood glucose, FPG** |  |  |  |
| number of participants, n | 19414 | 334 | 62 |
| prevalence of offspring CHD, n (%) | 485 (2.5) | 8 (2.4) | 6 (9.7) |

BMI, body mass index; CHD, congenital heart disease; CVH, cardiovascular health; FPG, fasting plasma glucose; PA, physical activity; TC, total cholesterol.

**Supplemental Table S2.** **The association of maternal overall cardiovascular health (CVH) with congenital heart disease (CHD) in offspring**

| **Materna CVH** | **Offspring CHD, No./total No. (%)** | **Relative risk (95% CI)*** | | |
| --- | --- | --- | --- | --- |
|  |  | **Model 1: unadjusted** | **Model 2: adjusted** | **Model 3: IPTW** |
| **CVH categories** |  |  |  |  |
| **High** | 184/7846 (2.35) | 0.61 (0.43,0.86） | 0.64 (0.45,0.90） | 0.66 (0.47,0.93） |
| P value |  | 0.0043 | 0.0094 | 0.0188 |
| **Moderate** | 276/10949 (2.52) | 0.66 (0.47,0.91） | 0.67 (0.48,0.93） | 0.68 (0.49,0.95） |
| P value |  | 0.0121 | 0.0168 | 0.0251 |
| **Low** | 39/1015 (3.84) | 1 (reference) | 1 (reference) | 1 (reference) |
| **CVH score** |  |  |  |  |
| **Every one-point increase** | 499/19810 (2.52) | 0.94 (0.89,1.00） | 0.95 (0.90-1.01) | / |
| P value |  | 0.0507 | 0.1040 | / |

CHD, congenital heart disease; CI, confidence interval; CVH, cardiovascular health; IPTW, inverse probability of treatment weighting.

* The relative risk is computed from the log-binominal regression model, indicating the risk of offspring CHD in pregnant women of better CVH compared to the low CVH. Model 2 is adjusted for confounding variables including maternal demographics, drinking, medication use, previous pregnancies, and chronic diseases; Model 3 addresses all the confounding listed in the method by the IPTW method.

**Supplemental Table S3.** The population attributable fraction of maternal cardiovascular health (CVH) status on congenital heart disease (CHD) in offspring

| **Exposure: CVH level** | **% of population** | **Adjusted RR** | **Population attributable fraction (%)*** |
| --- | --- | --- | --- |
| High | 39.61 | 1.00 | - |
| Moderate | 55.27 | 1.05 (0.87, 1.27) | 2.63 |
| Low | 5.12 | 1.57 (1.12, 2.21) | 1.86 |
| **All** | 100.0 |  | 4.49 |

CVH, cardiovascular health; RR, relative risk.

*The formula for multicategory exposure is
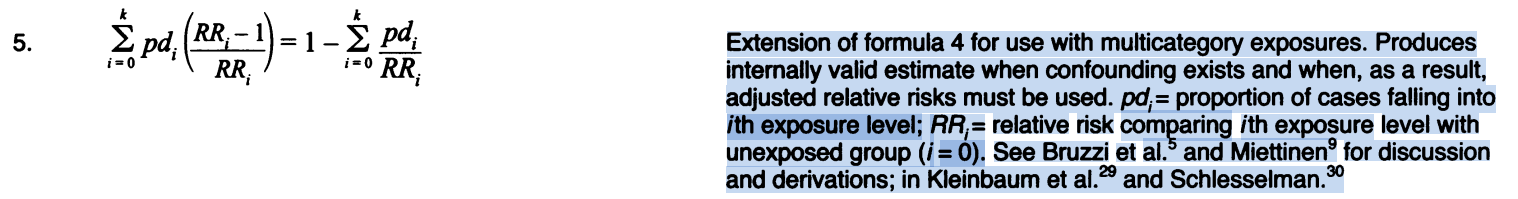
, considering for possible confounding in cohort study. The RR is computed with the high CVH as reference.

**Supplemental Table S4.** **The association of individual maternal cardiovascular health (CVH) metrics with congenital heart disease (CHD) in offspring**

|  | **Offspring CHD,**  **No./total No. (%)** | **Model 1: only adjusted for other CVH metrics** | | **Model 2: fully adjusted** | |
| --- | --- | --- | --- | --- | --- |
|  |  | **Relative risk**  **(95% CI)** | **P value** | **Relative risk (95% CI)** | **P value** |
| **PA** |  |  |  |  |  |
| ideal | 21/714 (2.9) | 1.19 (0.78,1.84） | 0.4207 | 1.22 (0.79,1.88） | 0.3672 |
| non-ideal | 478/19096 (2.5) | 1 (reference) |  | 1 (reference) |  |
| **Smoke** |  |  |  |  |  |
| ideal | 492/19479 (2.5) | 1.21 (0.58,2.54） | 0.6102 | 1.29 (0.61,2.74） | 0.5027 |
| non-ideal | 7/331 (2.1) | 1 (reference) |  | 1 (reference) |  |
| **Sleep health** |  |  |  |  |  |
| ideal | 355/13975 (2.5) | 1.03 (0.85,1.24） | 0.7779 | 1.06 (0.87,1.28） | 0.5835 |
| non-ideal | 144/5835 (2.5) | 1 (reference) |  | 1 (reference) |  |
| *Sleep duration* | | 0.92 (0.84,1） | **0.0482** | 0.91 (0.83,0.99） | **0.0243** |
| **BMI** |  |  |  |  |  |
| ideal | 291/11956 (2.4) | 0.93 (0.78,1.11） | 0.4029 | 0.95 (0.79,1.13） | 0.5506 |
| non-ideal | 208/7854 (2.7) | 1 (reference) |  | 1 (reference) |  |
| *BMI values* |  | 1.03 (1,1.06） | **0.0395** | 1.04 (1.01,1.07） | **0.0184** |
| **BP** |  |  |  |  |  |
| ideal | 322/13007 (2.5) | 0.98 (0.82,1.18） | 0.8233 | 1 (0.83,1.19） | 0.9581 |
| non-ideal | 177/6803 (2.6) | 1 (reference) |  | 1 (reference) |  |
| *SBP values* |  | 1 (0.99,1.01） | 0.8071 | 1 (0.99,1.01） | 0.6678 |
| *DBP values* |  | 1 (0.98,1.01） | 0.733 | 1 (0.98,1.01） | 0.6762 |
| **Blood lipid** |  |  |  |  |  |
| ideal | 374/15946 (2.4) | 0.73 (0.60,0.89） | **0.002** | 0.73 (0.59,0.89） | **0.0018** |
| non-ideal | 125/3864 （3.2) | 1 (reference) |  | 1 (reference) |  |
| *TC values* |  | 1.01 (1,1.01） | **0.0014** | 1.01 (1,1.01） | **0.001** |
| **Blood glucose** |  |  |  |  |  |
| ideal | 485/19414 (2.5) | 0.73 (0.43,1.23） | 0.2401 | 0.73 (0.43,1.24） | 0.2445 |
| non-ideal | 14/396 (3.5) | 1 (reference) |  | 1 (reference) |  |
| *FPG values* |  | 1.01 (1,1.02） | **0.021** | 1.01 (1,1.02） | **0.0124** |

BMI, body mass index; CHD, congenital heart disease; CVH, cardiovascular health; DBP, diastolic blood pressure; FPG, fasting plasma glucose; HDL, high density lipoprotein; PA, physical activity; SBP, systolic blood pressure; TC, total cholesterol.

**Supplemental Table S5. The association of maternal cardiovascular health (CVH) with congenital heart disease (CHD) in offspring (measured by logistic models)**

| **Materna CVH** | **Offspring CHD, No./total No. (%)** | **Odds ratio (95% CI)*** | | |
| --- | --- | --- | --- | --- |
|  |  | **Model 1: unadjusted** | **Model 2: adjusted** | **Model 3: IPTW** |
| **High** | 184/7846 (2.4) | 0.60 (0.42, 0.85) | 0.63 (0.44, 0.89) | 0.65 (0.46, 0.93) |
| P value |  | 0.0046 | 0.0097 | 0.0193 |
| **Moderate** | 276/10949 (2.5) | 0.65 (0.46, 0.91) | 0.66 (0.47, 0.93) | 0.67 (0.48, 0.95) |
| P value |  | 0.0125 | 0.0173 | 0.0258 |
| **Low** | 39/1015 (3.8) | 1 (reference) | 1 (reference) | 1 (reference) |

CHD, congenital heart disease; CI, confidence interval; CVH, cardiovascular health; IPTW, inverse probability of treatment weighting.

* The odds ratio is computed from the logistic regression model, indicating the odds of offspring CHD in pregnant women of better CVH compared to the low CVH. Model 2 is adjusted for confounding variables including maternal demographics, health information, and pregnancy history; Model 3 addresses the confounding by IPTW method.

**Supplemental Table S6. The association of maternal cardiovascular health (CVH) with congenital heart disease (CHD) in offspring (non-high density lipoprotein cholesterol as lipid metric)**

| **Materna CVH** | **Offspring CHD, No./total No. (%)** | **Relative risk (95% CI)*** | | |
| --- | --- | --- | --- | --- |
|  |  | **Model 1: unadjusted** | **Model 2: adjusted** | **Model 3: IPTW** |
| **High** | 184/7737 (2.4) | 0.66 (0.47, 0.92） | 0.68 (0.49, 0.96） | 0.71 (0.51, 0.99） |
| P value |  | 0.0134 | 0.0258 | 0.0461 |
| **Moderate** | 274/10940 (2.5) | 0.69 (0.5, 0.96） | 0.70 (0.51, 0.97） | 0.72 (0.52, 1） |
| P value |  | 0.0253 | 0.0331 | 0.0481 |
| **Low** | 41/1133 (3.6) | 1 (reference) | 1 (reference) | 1 (reference) |

CHD, congenital heart disease; CI, confidence interval; CVH, cardiovascular health; IPTW, inverse probability of treatment weighting.

* The relative risk is computed from the log-binominal regression model, indicating the risk of offspring CHD in pregnant women of better CVH compared to the low CVH. Model 2 is adjusted for confounding variables including maternal demographics, health information, and pregnancy history; Model 3 addresses the confounding by IPTW method.

**Supplemental Table S7. The association of maternal cardiovascular health (CVH) with congenital heart disease (CHD) in offspring (excluding the PA metric)**

| **Materna CVH** | **Offspring CHD, No./total No. (%)** | **Relative risk (95% CI)*** | | |
| --- | --- | --- | --- | --- |
|  |  | **Model 1: unadjusted** | **Model 2: adjusted** | **Model 3: IPTW** |
| **High** | 218/9526 (2.3) | 0.46 (0.30, 0.71） | 0.48 (0.31, 0.74） | 0.49(0.26, 0.95） |
| P value |  | 0.0005 | 0.0009 | 0.035 |
| **Moderate** | 259/9837 (2.6) | 0.53 (0.35, 0.82） | 0.54 (0.35, 0.83） | 0.55(0.28, 1.06） |
| P value |  | 0.0039 | 0.0048 | 0.0728 |
| **Low** | 22/447 (4.9) | 1 (reference) | 1 (reference) | 1 (reference) |

CHD, congenital heart disease; CI, confidence interval; CVH, cardiovascular health; IPTW, inverse probability of treatment weighting.

* The relative risk is computed from the log-binominal regression model, indicating the risk of offspring CHD in pregnant women of better CVH compared to the low CVH. Model 2 is adjusted for confounding variables including maternal demographics, health information, and pregnancy history; Model 3 addresses the confounding by IPTW method.
